# Protein synthesis is essential not only for consolidation but also for maintenance and post-retrieval reconsolidation of acrobatic motor skill in rats

**DOI:** 10.1186/1756-6606-2-12

**Published:** 2009-05-28

**Authors:** Ji-Yun Peng, Bao-Ming Li

**Affiliations:** 1 Institute of Neurobiology and State Key Laboratory of Medical Neurobiology, Institutes of Brain Science, Fudan University, Shanghai 200032, PR China; 2 Institute of Life Science, Nanchang University, Nanchang 330031, PR China

## Abstract

It has been reported that consolidation of motor skill, a type of non-declarative memories, requires protein synthesis, as hippocampus-dependent declarative memory does. However, little is known about the importance of protein synthesis in maintenance and especially post-retrieval reconsolidation of acrobatic motor skill. Here, we show that protein synthesis is essential not only for the consolidation but also for the maintenance and reconsolidation of a rotarod-running skill. Intra-ventricle infusion of the protein synthesis inhibitor anisomycin 0 h but not 2 h post-training caused a severe deficit in the acquisition of the rotarod-running skill. Protein synthesis inhibition (PSI) also caused a deficit in the maintenance of the rotarod-running skill, as well-trained rats demonstrated a deficit in the rotarod-running performance upon treatment with anisomycin. Similarly, PSI impaired the post-retrieval reconsolidation of the rotarod-running skill: well-trained rats treated with anisomycin 0 h but not 0.5, 2 and 4 h after the task performance exhibited amnesia for the running skill later on. Interestingly, rats treated with anisomycin 6 and 12 h post-retrieval exhibited amnesia for the running skill. Thus, protein synthesis is essential not only for the consolidation but also for the maintenance and post-retrieval reconsolidation of rotarod-running acrobatic motor skill.

## Introduction

It is known that consolidation and post-retrieval reconsolidation of hippocampus- or amygdala-dependent memories requires new protein synthesis [1-5]. Previous studies suggest that acquisition or consolidation of motor skills also requires protein synthesis [6,7]. For example, local infusion of anisomycin, a widely-used protein synthesis inhibitor, into the motor cortex immediately after training severely impaired the ability of rats to learn a reaching task [6]. Pre-training intra-peritoneal administration of cycloheximide, a relatively new protein synthesis inhibitor, blocked the between-session improvement of performance on an acrobatic motor skill [7].

Practice-induced improvement of motor skill occurs within and between training sessions [8-10], reflecting that there are two temporal phases for motor skill acquisition: one is fast and the other is slow [9]. As described above, acquisition or consolidation of motor skills requires new protein synthesis. However, it is unclear if maintenance and post-activation reconsolidation of motor skills also requires protein synthesis. To address this question, we investigated the effects of protein synthesis inhibition (PSI) on the consolidation, maintenance and reconsolidation of an acrobatic motor skill in rats running on a rotating rod. It has been reported that intra-ventricle infusion of anisomycin with a dose of 100 μg/μl produces > 90% PSI in the brain at 10 min post-infusion and this inhibition lasts about one hour, indicating a narrow time window for anisomycin to produce effect [1]. Thus, we selected the 100 μg/μl dose of anisomycin for intra-ventricle administration to evaluate the impacts of global PSI in the brain on the rotarod-running skill.

## Results

### Consolidation of rotarod-running skill requires protein synthesis

We first investigated the impact of PSI on the consolidation of the rotarod-running skill. In this experiment, naïve rats were trained one trial each day for 3 consecutive days. Anisomycin (100 μg in 1 μ1 ACSF) was infused into the left and right lateral ventricles at 0, 2, 6 or 12 h after each daily trial. ACSF was infused as vehicle control. Retention was tested on day 5 and 6, one trial each day.

**Figure 1:**
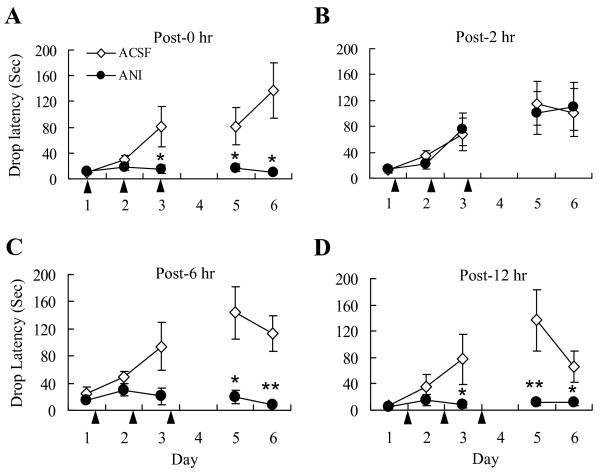
**Consolidation of rotarod-running skill requires protein synthesis**. Naïve rats were trained one trial each day for 3 consecutive days. Anisomycin or ACSF was infused bilaterally into the ventricle at 0, 2, 6 or 12 h after each trial, as indicated by the black arrows in A, B, C and D, respectively. Retention was tested on day 5 and 6, one trial each day. Data are expressed as means ± SEM. (A) Rats treated with anisomycin (ANI) 0 h post-trial showed a deficit in acquisition and retention of the rotarod-running skill. * p < 0.05 for ANI group (n = 9 rats) vs. ACSF group (n = 7 rats), unpaired t-test. (B) Rats treated with anisomycin (ANI) 2 h post-trial performed the rotarod-running task equally well with those treated with ACSF. ANI group, n = 10 rats; ACSF group, n = 9 rats. (C) Rats treated with anisomycin (ANI) at 6 h post-training showed a deficit in acquisition and retention of the rotarod-running skill. * p < 0.05, ** p < 0.01 for ANI group (n = 8 rats) vs. ACSF group (n = 10 rats), unpaired t-test. (D) Rats treated with anisomycin (ANI) 12 h post-trial showed a deficit in acquisition and retention of the rotarod-running skill. * p < 0.05, ** p < 0.01 for ANI group (n = 7 rats) vs. ACSF group (n = 8 rats), unpaired t-test.

As shown in Figure 1A, the rats treated with ACSF at 0 h post-trial gradually improved performance during the 3-day's training, and performed the task quite well in the testing trials on day 5 and 6, indicating that the animals had acquired the rotarod-running skill. However, the rats treated with anisomycin 0 h post-trial failed to acquire the running skill: their performance was not improved with training and was correspondingly poor on the testing trials. It is reported that a high dose anisomycin would produce side effects including motor inability [11]. However, the rats treated with anisomycin 2 h post-trial perfectly acquired the rotarod-running skill, as the ACSF controls did (Figure 1B), indicating that the dose of anisomycin we used did not produce any motor inability. These results suggest that the consolidation of the rotarod-running skill requires protein synthesis, with a post-training time window less than 2 hours, consistent with previous studies [6,7].

Interestingly, the rats treated with anisomycin 6 or 12 h post-trial also demonstrated a severe deficit in the acquisition of the rotarod-running skill and consequently exhibited a poor performance in the retention trials (Figure 1C and 1D). It was likely that the consolidation of the motor skill includes a long-lasting late phase that is sensitive to PSI, or the maintenance of the motor skill was interrupted upon PSI.

### Maintenance of rotarod-running skill requires protein synthesis

**Figure 2:**
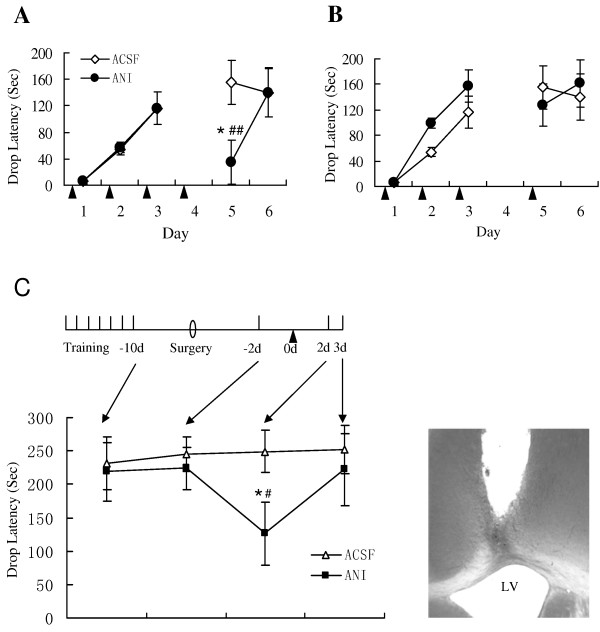
**Maintenance of rotarod-running skill requires protein synthesis**. (A) Naïve rats were trained one trial each day for 3 consecutive days. Anisomycin or ACSF was infused bilaterally into the lateral ventricles 6 h pre-trial. An additional ANI infusion was performed on day 4, with no training after the infusion. Rats treated with anisomycin (ANI) performed the rotarod-running task equally well with those treated with ACSF in the 3 training days. The performance level of ANI-treated rats dropped dramatically in the day-5 retention test, but was recovered in the day-6 retention trial. * p < 0.05, for ANI group (n = 9 rats) vs. ACSF group (n = 12 rats), unpaired t-test. ^## ^p < 0.01, for ANI group on day 3 vs. day 5, paired t-test. Data are expressed as means ± SEM. (B) Naïve rats were trained one trial each day for 3 consecutive days. Anisomycin or ACSF was infused bilaterally into the lateral ventricles 6 h pre-trial. An additional ANI infusion was performed on day 5, 6 hours prior to the retention test. Rats treated with anisomycin (ANI) performed the rotarod-running task equally well with those treated with ACSF in the 3 training days. The performance level of ANI-treated rats was similar to that of the ACSF group in the day-5 and day-6 retention trials. ANI group, n = 9 rats; ACSF group, n = 12 rats. Data are expressed as means ± SEM. (C) Naïve rats were trained one trial each day for 7 consecutive days. Surgery was performed on day 4 after the last training trial. Rats were retrained one trial on day 4 after the surgery. Anisomycin (ANI) or ACSF was administered into the lateral ventricles 2 days post-retraining, as indicated by the black arrow, and were tested one trial each day for the subsequent 2 days. The two groups performed the task equally well in the last training trial and did so in the post-surgery re-training trial. However, the anisomycin group exhibited a deficient performance in the 1^st ^testing trial. The deficient performance was recovered in the 2^nd ^testing trial. * p < 0.05 for ANI group (n = 7 rats) vs. ACSF group (n = 7 rats), unpaired t-test; ^# ^p < 0.05 for post-infusion trial vs. pre-infusion trial, paired t-test. Data are expressed as means ± SEM. *Right bottom*: A representative placement of the guide cannuale for anisomysin infusion. The trace for the injection needle could be identified clearly. LV: lateral ventricle.

To investigate if the maintenance of the rotarod-running skill involves protein synthesis, we bilaterally administered anisomycin (100 μg in 1 μ1 ACSF) into the ventricles 6 h before each daily trial for 3 consecutive days. An additional infusion was carried out on day 4. Retention trials were performed on day 5 and 6. As shown in Figure 2A, the anisomycin group well acquired the rotarod-running skill through the training, as the ACSF controls did. Surprisingly, the anisomycin group demonstrated a deficient performance on day 5 and this deficit was quickly rescued on day 6, indicating that the treatment with anisomycin on day 4 interfered with the maintenance of the rotarod-running skill.

We then conducted a similar experiment as in Figure 2A, except that the additional anisomycin treatment was performed 6 h prior to the trial on day 5 (Figure 2B). In Figure 2A, the interval between the anisomycin treatment and the motor skill test was 30 hours, whereas that in Figure 2B was 6 hours. As shown, the anisomycin group performed normally in the test trial on day 5. Taken together, these results suggest that PSI-induced interference with the maintenance of the rotarod-running skill needs a relatively long time to develop. That is to say, practice in time could prevent the rotarod-running skill from being interrupted by PSI.

In order to further demonstrate that the maintenance of the rotarod-running skill involves a sustained protein synthesis, we then conducted the following experiment with a different protocol. Naïve rats were trained on the rotarod-running task one trial each day for 7 consecutive days in order for the animals to reach a well-trained level. Surgery for cannula implantation was performed on day 4 after the last training trial. Rats were given 4 days for recovery and then received a one-trial retraining. Anisomycin (100 μg in 1 μ1 ACSF) was bilaterally administered into the ventricles 2 days post-retraining. ACSF was infused as vehicle control. The animals were tested one trial each day for the subsequent 2 days (Figure 2C). As shown, the anisomycin and ACSF groups performed the task equally well in the last training trial and did so in the post-surgery re-training trial. However, the anisomycin group exhibited a deficient performance in the 1^st ^testing trial. As the interval between the anisomycin administration and the training was 10 days and that between the drug treatment and the retraining trial was 2 days, the deficit can not be explained as a result of deficient consolidation. This result strongly indicates that the maintenance of the rotarod-running skill requires a sustained protein synthesis and is therefore sensitive to PSI. Consistent with the result shown in Figure 2A, the deficient performance was quickly rescued in the next retention trial.

### Reconsolidation of rotarod-running skill requires protein synthesis

It is well known that hippocampus- or amygdala-dependent declarative memory becomes labile after it is retrieved and undergoes a reconsolidation involving protein synthesis [3,4]. Here, we investigated if protein synthesis is also required for post-retrieval reconsolidation of the rotarod-running skill.

**Figure 3:**
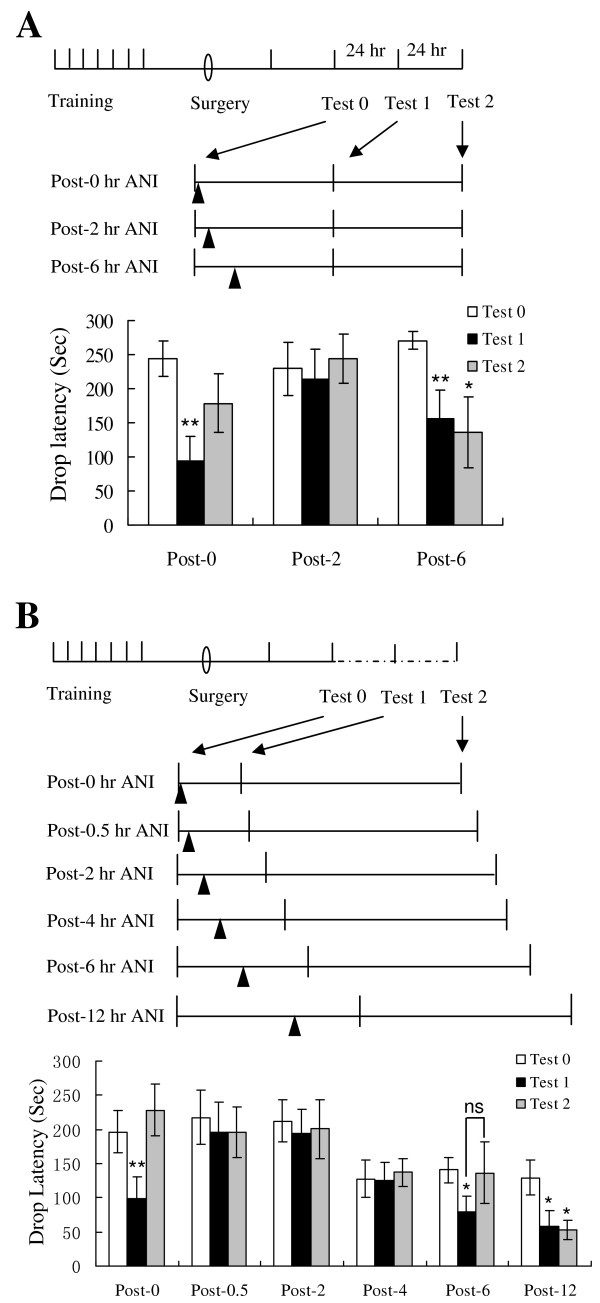
**Reconsolidation of rotarod-running skill requires protein synthesis**. Naïve rats were trained one trial each day for 7 consecutive days. Surgery was performed on day 4 after the last training trial. Rats were retrained one trial on day 4 after the surgery, and then received a retrieval trial (Test 0) to reactivate the running-skill memory. (A) Rats were infused with anisomycin at 0, 2 or 6 h after Test 0, respectively. They were tested one trial each day for the subsequent 2 days (Test 1 and Test 2). Rats in post-0 group (n = 9) exhibited a deficient performance in Test 1 and the deficit was partially recovered in Test 2. Rats in post-2 group (n = 7) exhibited a normal performance in both Test 1 and 2. Rats in post-6 group (n = 8) showed a deficit in the task performance in both Test 1 and 2. * p < 0.05, **p < 0.01 vs. Test 0, paired t-test. (B) Rats were infused with anisomycin at 0, 0.5, 2, 4, 6, or 12 h after Test 0, respectively. They received a retention test at 6 h after anisomycin infusion (Test 1) and a second retention trial 24 h later (Test 2). Rats in post-0, post-6, and post-12 groups exhibited an impaired performance in Test 1. The deficient performance of the post-0 group was recovered in Test 2. * p < 0.05 vs. Test 0, paired t-test. ** p < 0.01 vs. Test 0, paired t-test. ns: not significant. n = 12, 8, 8, 9, 8, 7 rats for the post-0, post-0.5, post-2, post-4, post-6 and post-12 groups, respectively. Data are expressed as means ± SEM.

To do this, we trained naïve rats on the rotarod-running task one trial each day for 7 consecutive days. Surgery for cannula implantation was carried out on day 4 after the last training trial. Rats were given 4 days for recovery and then received a one-trial re-training. One day later, rats received a retrieval trial to reactivate the running-skill memory (Test 0). The animals were tested one trial each day for the subsequent 2 days (Test 1 and Test 2). The animals were given anisomycin at 0, 2 or 6 h after the retrieval trial, respectively. Anisomycin (100 μg in 1 μ1 ACSF) was bilaterally infused into the lateral ventricles (Figure 3A).

As shown, the rats treated with anisomycin 0 h post-retrieval exhibited a deficient performance in Test 1 and this deficit was partly rescued in Test 2, while those treated with anisomycin 2 h post-retrieval performed the task normally in Test 1 and 2, suggesting that the well-learned rotarod-running skill becomes unstable after reactivation and undergoes a protein-synthesis-dependent reconsolidation.

The rats treated with anisomycin 6 h post-retrieval also exhibited a deficient performance in Test 1, and this deficit was not rescued in Test 2 (Figure 3A). This result was similar with the result in Figure 1C showing that the animals administered with anisomycin 6 h post-training failed to acquire the rotarod-running skill. It might be possible that this deficit was a result of PSI-induced interference with the maintenance of the motor skill, as we already showed that the maintenance of the rotarod-running skill requires a sustained protein synthesis (see Figure 2), or the deficit was because of the deficiency of the late phase reconsolidation.

To address this issue, we used a modified experimental protocol (Figure 3B), in which six time points (0, 0.5, 2, 4, 6 and 12 h) after the retrieval trial (Test 0) were selected for administration of anisomycin. Retention was tested 6 h after anisomycin treatment (Test 1). 24 h later, a second retention trial was performed (Test 2). The 6-h interval between the anisomycin treatment and the 1^st ^retention trial was selected as this arrangement did not affect the maintenance of the rotarod-running skill (see Figure 2B).

As shown in Figure 3B, the post-0 h, post-6 h and post-12 h groups of rats exhibited a severe deficit in the rotarod-running performance in Test 1, while the post-0.5 h, post-2 h and post-4 h groups of rats performed the task normally. In Test 2, the post-0 h group showed a fully-recovered performance, whereas the post-12 h group did not at all. For the post-6 group, four of the eight rats recovered to normal level and the remaining four rats did not. These results were well consistent with those shown in Figure 3A, again indicating that the well-learned rotarod-running skill becomes unstable after reactivation and undergoes a protein-synthesis-dependent reconsolidation, which includes an early- and a late-phase processes. The early-phase reconsolidation has a narrow PSI-sensitive time window, whereas the late-phase reconsolidation begins several hours post-retrieval and has a much wider PSI-sensitive time window.

## Discussion

Our present results are consistent with previous studies showing that consolidation of motor skill requires protein synthesis. More importantly, we provide evidence for the first time that maintenance and post-performance reconsolidation of motor skill also involve protein synthesis.

### PSI-sensitive consolidation of the rotarod-running skill

The present study showed that infusion of anisomycin into the lateral ventricles 0 h but not 2 h after each daily trial severely impaired the between-trial improvement of the rotarod-running skill. This result was consistent with the previous study by Luft et al. (2004) showing that intra-peritoneal administration of cycloheximide 1 h pre-training produced a deficit in the between-session improvement of performance on an acrobatic motor skill [7]. Thus, between-trial consolidation of the rotarod-running skill occurs within a 2-h time window and is dependent on protein synthesis.

Interestingly, administration of anisomycin 6 h or 12 h post-trial also caused a deficit in the acquisition of the rotarod-running skill. This deficit would be due to the interruption with the maintenance of the motor skill under establishment. Indeed, our evidence showed that the maintenance of the well-established motor skill was sensitive to PSI (see Figure 2C). An alternative interpretation is that, the consolidation of the rotarod-running skill includes a PSI-sensitive late-phase process. Although we do not have a direct evidence for this interpretation, the existence of a PSI-sensitive late-phase reconsolidation of the motor skill (see Figure 3) strongly suggests such possibility.

### PSI-sensitive maintenance of the rotarod-running skill

In the present study, the well-trained animals exhibited a deficit in the rotarod-running skill after treated with anisomycin (Figure 2C). The deficient performance could not be explained as a deficit in the consolidation of the original training because the drug was administered several days post-training, nor as a deficit in post-retrieval reconsolidation because the anisomycin-treated animals were not required to perform the task at the time when the drug was administered. Thus, it was most likely that protein synthesis inhibition disrupted the maintenance of the already-established running skill.

Motor skill memory is different from hippocampus- and/or amygdala-dependent memory in that, the latter becomes stable after well consolidated and is resistant to protein synthesis inhibition, given that it is not activated. It is possible that the motor structures responsible for motor-skill memory undergo dynamic reorganization in their fine structures in order to maintain the skill, a process that requires continuous protein synthesis. For example, Kleim et al. (2003) reported that functional organization of adult motor cortex is dependent upon a continued protein synthesis [12].

Interestingly, the interference needed quite a long time to develop (see Figure 2A and 2B). It is possible that the dynamic reorganization of motor structures is slow and gradual. A transient PSI would produce an error in the reorganization, which does not produce an immediate deficit (Figure 2B: normal functional manifestation 6 h post-PSI), probably because synapse loss could not happen immediately, but finally result in functional loss (Figure 2A: disfunction 30 h post-PSI).

One may argue that, if the maintenance of the motor skill is dependent on continuous protein synthesis, how it can be explained that the ANI-injection 6 h prior to the first retrieval trial on day 5 (Figure 2B) did not affect the performance on the second retrieval trial on day 6? A possible explanation is that, the retrieval trial on day 5 prevented the motor system from being further affected by the PSI. Thus, performance on the second trial on day 6 remained normal.

### PSI-sensitive reconsolidation of the rotarod-running skill

It is known that hippocampus- or amygdala-dependent declarative memory becomes unstable after reactivation and undergoes a protein-synthesis dependent reconsolidation [2,4]. In the present study, we found that there were two separate PSI-sensitive time windows after the rotarod-running skill was reactivated. The early window was quite narrow, reflecting a fast process, and the late one started about 4–6 hours later, lasting over 6 hours. The deficit induced by protein synthesis inhibition during the early window could be rescued by a single-trial practice, whereas the PSI-induced deficit during the late window could not be rescued, suggesting that the protein-synthesis dependent late-phase reconsolidation of the motor skill starts slowly but lasts for a long time. It is possible that, like hippocampus- and amygdala-dependent declarative memory, motor-skill memory may also involve renewal of the existed neural circuits after it is re-activated [4].

### Requirement of a long-lasting late phase for consolidation/reconsolidation

Experience-driven improvement on motor performance occurs within and between practice sessions. The within-session improvement involves a process that selects and establishes an optimal strategy or plan for the performance of a task [9]. A rapid change in neural plasticity would occur within this phase, and neural signaling initiated here would trigger a late-phase consolidation. It is thought that the late-phase consolidation involves structural modifications of motor structures and strengthening of synaptic links between neurons in the structures, and therefore needs a relatively long time to develop [9,12]. It has been suggested that the between-session improvement appears at least several hours later [13,14]. Consistently, the present study showed that the consolidation and reconsolidation of the rotarod-running skill was disrupted by PSI introduced even 12 hours after training/retrieval.

The early-phase consolidation/reconsolidation might share a common mechanism as hippocampus- and/or amygdala-dependent memory does, although the brain structures involved are different. For hippocampus- and/or amygdala-dependent memory, many molecules are required for the memory to be consolidated to a long-term form, such as PKA signaling cascades, ERK/MAPK signaling pathways, gene transcription and protein synthesis [3,4,15,16].

Different from hippocampus- and/or amygdala-dependent memory, motor-skill memory is difficult to be acquired and requires quite many repetitions and long time to evolve. However, once established, motor-skill memory is difficult to be forgotten. The present study showed that consolidation and reconsolidation of the rotarod-running skill required a long-lasting PSI-sensitive late phase. This result suggests that there must be quite different mechanisms that underlie late-phase consolidation/reconsolidation of the motor skill at molecular, cellular and system levels. The result that PSI-induced deficit during late-phase consolidation/reconsolidation was more difficult to be rescued suggests that, the neural modifications happening during this phase play a key role for the establishment of motor skill memory. Kleim et al. (2004) reported that motor skill learning produces protein synthesis dependent cortical synaptogenesis and motor-map reorganization, which occur during late but not early phase of learning [12,17].

### Action sites of intra-ventricularly administered anisomycin

As anisomycin administered via intra-ventricle infusion could spread and act throughout the central nervous system, we do not know exactly the target structures where anisomycin produces its effects. It is known that the motor cortex, cerebellum and basal ganglia play an essential role in acquisition and consolidation of motor skills [18]. It was possible that the motor cortex was a cortical area for anisomycin action. Luft et al. reported that anisomycin infused into the motor cortex immediately after training severely impaired the ability of rats to learn a reaching task [6]. Kleim et al. (2003) reported that functional organization of the motor cortex is dependent upon continued protein synthesis [12]. Kleim et al. (2004) reported that cortical synaptogenesis and motor-map reorganization occur during late, but not early, phase of motor skill learning [12,17]. The basal ganglia and cerebellum would also be the target structures for anisomycin action, as these two structures are involved in motor learning and performance by integrating error signals in their loop circuits with motor cortex [18,19].

## Methods

### Animals and Surgery

Male Spraque-Dawley rats (60–90 days old; 250–320 g) were used. Rats were purchased from the Shanghai Laboratory Animal Center, Chinese Academy of Sciences (Shanghai, China). They were housed 2–3 per cage under constant temperature (23 ± 1°C) and light-controlled vivarium (12 h light/12 h dark cycles). Food and water were available *ad libitum*. All experimental procedures involving the use of the animals were in accordance with the *Guide for the Care and Use of Laboratory Animals *issued by the National Institutes of Health, USA (NIH Publications No. 80-23; 1996), and were approved and monitored by the Ethical Committee of Animal Experiments at the Institute of Neurobiology, Fudan University (Shanghai, China).

Surgical procedures were performed under sodium pentobarbital anesthesia (40 mg/kg i.p.). Rats were restrained in a stereotaxic apparatus (SN-2; Narishige, Japan) and implanted bilaterally with guide cannula (stainless steel, 23 gauge), 1.5 mm above the lateral ventricle (using the coordinates of Panxinos and Watson's *the Rats Brain in Stereotaxic Coordinates*, 1986; Lateral ventricle AP -0.9 mm to bregam, ML 1.5 mm to the midsagittal suture line, and V 3.9 mm to the skull surface). The cannula were fixed in place with dental cement and secured with skull screws. A stylus was inserted into the guide cannula to prevent clogging and reduce the risk of infection. Rats were allowed a recovery period of 4–7 days before behavioral training or testing.

### Chemicals and Microinjection

Artificial cerebrospinal fluid (ACSF) was used as vehicle for anisomycin, which was composed of (in mM) 125 NaCl, 1.25 NaH_2_PO_4_, 2.5 CaCl_2_, 1.5 MgSO_4_, 26 NaHCO_3 _(pH 7.4). Anisomycin (Sigma Chemical Company, St. Louis, Missouri, USA) was dissolved in 1 N HCl, adjusted to pH 7.2 and diluted to the final concentration of 100 μg/μl.

For intra-ventricle infusion of anisomycin or vehicle, rats were held manually, the stylus was removed from the guide cannula, and a 30-gauge injection needle was carefully inserted into the guide cannula. The injection needle extended 1.5 mm from the tip of the guide cannula, targeting at the lateral ventricle. Injection was done at a rate of 1.0 μl/min and the injection needle was left in place for additional 2 min after completion of injection. Bilateral injections were done simultaneously.

### Rotarod-running task

Rats were trained on a rotarod-running task. The rod was a plastic bar with 6.0 cm in diameter and was placed 60 cm above ground. Under the rotarod was there a cage used to protect an animal dropping from the rod. The rotating speed of the rod could be adjusted manually. All rats received a 5-min adaptation training, in which the rod was rotated at very low speed (5 rpm) [20].

For training, rats were placed on the rod and its rotation speed was slowly adjusted from low to high and finally up to 40 rpm. Each trial lasted 5 min under a rotation speed of 40 rpm and each animal was trained one trial in a daily session. The time for a rat to keep running on the rod was recorded as drop latency. If a rat did not drop from the rod, its drop latency was recorded as 5 min (300 sec).

### Histology

Rats were anaesthetized with an overdose of sodium pentobarbital and transcardially perfused with 0.9% saline followed by 10% formalin. Their brains were removed from skulls, and then put in 10% glucose solution for several hours till sinking bottom, then transferred to 20% glucose solution, and finally sank in 30% glucose solution. The brains were coronally sectioned at 40-μm thickness. The brain sections were mounted on gelatin-subbed glass slides and stained with thionin for histological examination of infusion sites [21].

## Conclusion

In summary, the present study provides evidence that protein synthesis is essential not only for the consolidation but also for the maintenance and post-reactivation reconsolidation of acrobatic motor skill in rats.

## Competing interests

The authors declare that they have no competing interests.

## Authors' contributions

JYP carried out the experiments and drafted the manuscript. BML supervised the study, participated in its design, and revised the manuscript. All authors read and approved the final manuscript.
